# Different Chinese patent medicine therapies for migraine: A protocol for systematic review and network meta-analysis

**DOI:** 10.1097/MD.0000000000024179

**Published:** 2021-01-15

**Authors:** Liangen Hu, Zhangren Yan, Feng Chen, Wei Xiao, Liting Liu, Chunhua Huang

**Affiliations:** a Xinyu Hospital of Traditional Chinese Medicine, Xinyu; b Affiliated Hospital of Jiangxi University of Traditional Chinese Medicine; c Jiangxi University of Traditional Chinese Medicine, Nanchang, Jiangxi Province, China.

**Keywords:** Chinese patent medicine, migraine, network meta-analysis, protocol

## Abstract

**Background:**

Migraine is a clinically high incidence rate of neurovascular disease. It is a recurrent headache. It is characterized by nausea, vomiting, fear of voice, and photophobia. Nowadays, a large number of randomized controlled clinical studies have shown that Chinese patent medicine has the advantages of good curative effect and high safety in the treatment of migraine. However, due to the variety of proprietary Chinese medicines, their relative effectiveness and safety have not yet been verified. Therefore, this study will use the network meta-analysis method to verify the effectiveness and safety of different kinds of Chinese patent medicines in the treatment of migraine.

**Methods:**

All randomized controlled trials of Toutongning capsule, Yangxue Qingnao granule, naoxintong capsules, Tianmagouteng granules in the treatment of migraine were searched from PubMed, Cochrane Library, web of science, EMBASE, sinomed, CNKI, Wanfang database, VIP. The retrieval time is from the establishment of the database to November 18, 2020. In order to avoid omission, we will manually retrieve relevant references and conference papers. According to the inclusion and exclusion criteria, we evaluated the quality and risk of all the retrieved literatures. Methodological quality assessment and bias risk will be assessed using the Cochrane bias risk tool. Revman 5.3, WinBUGS 1.4.3, and stata14.2 software will be used for all data analysis.

**Results:**

This study will directly or indirectly compare the effectiveness of different interventions on migraine outcome indicators, and rank the effectiveness. The main outcome measures included total effective rate (total effective rate = rocovery + obvious effective + effective/total number of cases × 100%), visual analogue scale (VAS) score, and secondary outcome indicators included analgesic effect evaluation index and quality of life scale.

**Conclusion:**

To provide evidence for evidence-based medicine and clinical researchers to choose more effective Chinese patent medicines to treat migraine.

## 1 Introduction

Migraine is a clinically high incidence rate of neurovascular disease. Women are more than men. This disease is a type of primary headache. It often recurs, with severe headache, accompanied by nausea, vomiting, fear of voice, and photophobia. When migraine attacks occur, patients often fail to enter normal activities due to pain, which seriously affects the quality of life of patients.^[1]^ Based on the latest epidemiological update results, the global burden of migraine has increased greatly in the past 15 years. In the past 15 years, the frequency of migraine in women is higher than that in men, and the incidence age is between 15 and 49 years old.^[2]^ Western medicine treatment of the disease includes non-specific analgesic drugs such as ibuprofen, aspirin, etc, and specific painkillers such as sumatriptan and ergotamine. But these 2 kinds of drugs are often accompanied by serious adverse reactions, so the clinical is limited.^[3]^ Therefore, we urgently need to explore a more effective and safe treatment.

As a traditional Chinese medicine, traditional Chinese medicine (TCM) has been inherited for its remarkable efficacy and high safety. TCM has a history of thousands of years in China and has been gradually recognized by all countries in the world. Traditional Chinese medicine is believed to be effective in improving various diseases, including migraine. There are many ways to treat migraine in Chinese medicine, including Chinese herbal decoction, Chinese patent medicine, acupuncture, and massage. Chinese patent medicine is made of Chinese herbal medicines as raw materials and processed into various different dosage forms of Chinese medicine products, including pills, powders, ointments. Chinese patent medicines have the characteristics of convenience and effectiveness, good taste, easy storage, and carrying, which are favored by patients in clinical application. Migraine belongs to the category of “headache” in traditional Chinese medicine. According to traditional Chinese medicine, there are 2 main mechanisms of pain: one is “pain due to obstruction of Qi and blood in the meridians of the human body, which will cause various kinds of pain”; the other is “pain due to lack of Qi and blood or excessive consumption of Yin essence,” which refers to all kinds of pain caused by the loss of nourishment of meridians due to insufficient Qi and blood or excessive consumption of Yin essence. Traditional Chinese medicine treatment of headache mainly plays the role of promoting blood circulation, removing blood stasis and removing stagnation, nourishing blood, and smoothing liver, so as to relieve pain.^[3]^ However, due to the variety of types of Chinese patent medicines, including Toutongning capsule, Yangxueqingnao granule, Naoxintong capsule, Tianma Gouteng granule, Yanhu Zhitong dripping pills, the efficacy of each Chinese patent medicine is also different. The results of meta-analysis showed that Yangnaoqing granules could reduce the frequency of headache^[4]^; Toutongning capsule has a definite curative effect on migraine, and it is better than the western medicine group in terms of total effective rate, visual analogue scale (VAS) score, and improvement of hemorheological indexes.^[5]^ But all of these are just a single Chinese patent medicine versus Western medicine, and it is impossible to compare multiple Chinese patent medicines. As we all know, each Chinese patent medicine has different advantages in the treatment of diseases, so the choice of which treatment method to use has brought confusion to clinical operators. The network meta-analysis can make positive comparisons of various interventions. Therefore, we will use network meta-analysis to systematically compare the effectiveness and safety of different Chinese patent medicine interventions, and provide evidence-based medicine for clinical researchers.

## 2 Protocol registration

This system review program will strictly follow the system review and meta-analysis program (PRISMA-P) preferred report items for reporting.^[6]^ The system review program has been registered on the INPLASY website (the registration number is INPLASY2020120010). If there are any adjustments during the entire study period, we will promptly revise and update the detailed information in the final report.

## 3 Methods

### 3.1 Inclusion and exclusion criteria

#### 3.1.1 Study type

Randomized controlled trials (RCTs) based on different Chinese patent medicine therapies for migraine, the language is limited in Chinese and English. The following literatures will be excluded

(1) Non-RCTs, such as conference reports, literature reviews;
(2) Literatures besides Toutongning capsule, Yangxueqingnao granule, Naoxintong capsule, and Tianmagouteng granule, other Chinese patent medicine therapies were used;
(3) The experimental group and the control group contained other interference therapy;
(4) The latest one was selected for repeated detection and repeated publication;
(5) Literatures with unavailable data and full text;
(6) Literatures that do not include the outcome indicators included in this study.

#### 3.1.2 Participants

Patients diagnosed as migraine according to the internationally recognized diagnostic criteria, with clear curative effect criteria, and unlimited age, race, gender, and source of cases. However, the following patients will be excluded:

(1) Patients who are unwilling to receive TCM treatment,
(2) Patients with serious cardiovascular and cerebrovascular diseases and mental diseases,
(3) Pregnant or lactating women.

#### 3.1.3 Interventions

The experimental group was treated with Chinese patent medicine, including Toutongning capsule, Yangxueqingnao granule, Naoxintong capsule, and Tianmagouteng granule, while the control group was treated with western medicine alone. Both the experimental group and the control group could cooperate with conventional medical treatment.

#### 3.1.4 Outcome indicators

The main outcome measures included total effective rate, VAS score, and secondary outcome indicators included analgesic effect evaluation index and quality of life scale.^[7]^

### 3.2 Data sources and search strategies

**Figure 1:**
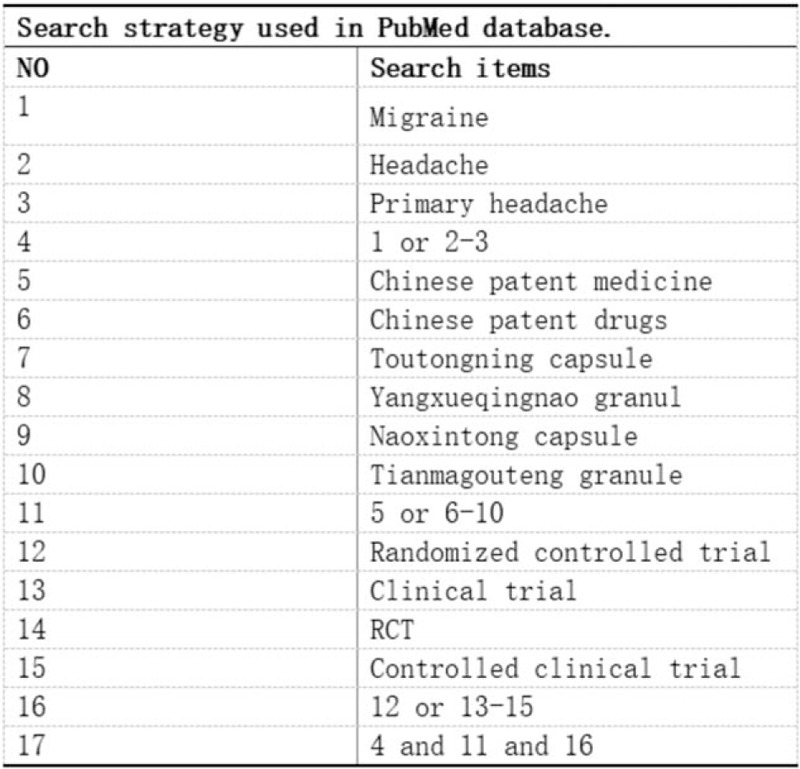
Search strategy used in PubMed database.

Computer retrieval was conducted in PubMed, Cochrane Library, Web of Science, Embase, SinoMed, CNKI, WanFang database, VIP. The retrieval period was until November 18, 2020. The search terms are: “ Toutongning capsule,” “Yangxueqingnao granule,” “Naoxintong capsule,” “Tianmagouteng granule,” “Chinese patent medicine,” “Chinese patent drugs,” “Migraine,” “headache,” “Primary headache.” The search strategy is to combine search terms with subject words and free words. The data retrieval strategy is shown in Figure 1.

### 3.3 Selection of studies and data extraction

First, 2 reviewers (LGH and ZRY) independently screened according to the inclusion and exclusion criteria of the literature, and then cross-checked. In case of disagreement, the third evaluator could make a decision. We will establish the document information extraction table in Excel. The extracted information includes: author, publication time, number of cases, allocation method, intervention measures, course of treatment, and outcome indicators.

### 3.4 Risk assessment of bias

Two reviewers independently assessed the bias risk of the articles included in this study according to the Cochrane evaluator bias risk assessment tool. The evaluation criteria include 7 items: selections bias, performance bias, detect bias, attrition bias, reporting bias, and other bias. The evaluation results were evaluated as “high risk,” “’low risk,” and “unclear risk.”^[8,9]^

### 3.5 Statistical analysis

Revman 5.3 software was used for bias evaluation and traditional meta-analysis. The outcome indicators were count data, odds ratio (or), mean difference (MD) for measurement data, and 95% confidence interval (95% CI) for effect. WinBUGS 1.4.3 and stata14.2 were used for network meta-analysis.^[10,11]^ In WinBUGS software, Markov chain Monte Carlo (MCMC) method is used for Bayesian network meta-analysis. Four chains are used for simulation. The number of iterations is set to 50,000, and the first 20,000 annealing times are used to eliminate the influence of initial value, and the step length is set to 10.^[12]^ At the same time, the potential scale reduced factor (PRSF) was used to evaluate the convergence of the results. When the PRSF was close to or equal to 1.00 (1.00 ≤ PRSF ≤ 1.05), the results showed good convergence and high reliability.^[13]^ At the same time, the value of Sucra (surface under the cumulative ranking curves, SUCRA) and the area under the curve of Sucra were calculated by Stata software, so as to rank the effects of various interventions. The value range was 0 to 100. The larger the value and the larger the area under the curve, the better the effect of the intervention measures was.

### 3.6 Assessment of inconsistency

Due to the large number of interventions involved in this study, in the evidence network of each outcome indicator, the closed loop formed by studies with direct evidence and indirect evidence needs to be tested for inconsistency through Stata software. The inconsistency factor (if) was calculated, and the inconsistency was judged by the size of if value and *P* value.^[14]^ If the if is close to 0, 95% CI starts at 0, and *P* > .05, the results of direct comparison and indirect comparison are consistent. At the same time, the node split model is used to determine whether there is local inconsistency in each node.^[15]^ If *P* > .05, consistency model is used; otherwise, inconsistency model is used. For the consistency model analysis results, the stability of the results can be tested by the inconsistent model, when the inconsistent model factors include 0 and the inconsistency standard deviation. When the random standard deviation of the consistent effect model is approximately equal to the standard deviation of the inconsistent model, the consistency model results are more stable and reliable.^[16,17]^

### 3.7 Heterogeneity, subgroup analysis, sensitivity analysis

The heterogeneity between trials was quantified by *I*^2^ and *P* values.^[18]^ For the test results with obvious heterogeneity, the source of heterogeneity should be analyzed. According to the different sources of heterogeneity, subgroup analysis can be carried out, such as treatment time, course of disease, basic disease, race, gender, age, and so on. If there is no clear source of heterogeneity, only descriptive analysis can be carried out. The purpose of sensitivity analysis is to eliminate low-quality research and different statistical models.^[19]^ Observe the heterogeneity of different tests, observe whether the combined results change after different treatments, and analyze the strength, reliability, and stability of the results.

### 3.8 Assessment of publication bias

If the outcome indicators included in study ≥10, we will use funnel plot to evaluate bias.^[20]^ If the funnel plot shows asymmetry or distribution difference, it indicates publication bias or small sample effect.

### 3.9 Ethics and dissemination

As this is a protocol for systematic review and network meta-analysis, all data in this study are from published studies and do not involve patients, so ethical approval is not required. The results of the study will be distributed to peer-reviewed journals and published at relevant conferences.

## 4 Discussion

In recent years, with the continuous development and improvement of traditional Chinese medicine, Chinese medicine has accumulated rich experience in the treatment of migraine. Chinese patent medicine not only has the characteristics of safe and effective Chinese herbal medicine, but also has the advantages of convenience, good taste, easy preservation, and carrying. More and more clinical randomized controlled trials show that different types of Chinese patent medicine have significant curative effect and little side effects in the treatment of migraine, which indicates that Chinese patent medicine is worthy of further clinical promotion in the treatment of migraine.^[21–24]^ The Standard meta-analysis can only make a single comparison, while the network meta-analysis can make a pairwise comparison of various interventions, We will use the method of network meta-analysis to compare several different Chinese patent medicine, and get the order of effectiveness and safety, so as to provide evidence-based medicine for clinical decision-makers.

## Author contributions

**Conceptualization:** Liangen Hu, Zhangren Yan.

**Methodology:** Zhangren Yan, Feng Chen, Liting Liu, Chunhua Huang.

**Project administration:** Wei Xiao.

**Software:** Liting Liu.

**Supervision:** Chunhua Huang.

**Writing – original draft:** Liangen Hu, Zhangren Yan.

**Writing – review & editing:** Liangen Hu, Chunhua Huang.

## Abbreviations

Abbreviations: RCTs = randomized controlled trials, TCM = traditional Chinese medicine, VAS = visual analogue scale.
